# An estimate of syphilis incidence in Eastern Europe

**DOI:** 10.7189/jogh.02.010402

**Published:** 2012-06

**Authors:** Liam J. Herbert, Stephen I. Middleton

**Affiliations:** Centre for Population Health Sciences and Global Health Academy, University of Edinburgh, Scotland, UK

## Abstract

**Aim:**

Eastern Europe experienced epidemic levels of syphilis after the collapse of the Soviet Union. Presently data are less comprehensive outside the European Union (EU) and European Free Trade Association (EFTA). This review aims to identify published papers with suitable data to estimate a regional burden of disease for syphilis in the 19 member countries of Eastern Europe.

**Methods:**

A systematic literature review was conducted to identify published data relating to syphilis incidence in Eastern Europe through Web of Knowledge, PubMed and Google Scholar databases in addition to the latest surveillance report from the European Center for Disease Prevention and Control. A total of 381 papers fitted our search criteria; 30 papers were subjected to full text analysis.

**Results:**

Seven papers were included in this study and provided useable data for 13 out of 19 member countries. There was a high level of heterogeneity observed in the incidence rates from the member countries. Gross, population weighted and geographically subdivided incidence rate estimates were carried out but the comprehensiveness of some of the included data is doubtful.

**Conclusions:**

Despite the limits of the data, the incidence of syphilis in Eastern Europe is still substantially larger than that observed in the EU15 countries. This indicates that efforts to control syphilis in Eastern Europe can be enhanced; however, such goals would require significant investment in infrastructure, technology and surveillance mechanisms.

Syphilis is a sexually transmitted disease caused by the bacterium *Treponema pallidum pallidum* [1]. If untreated the disease can cause mortality of >60% of cases, mainly due to the complications in the tertiary phase of the disease. Also important is vertical transmission, known as congenital syphilis that is associated with increased pregnancy failure rates and severe birth defects. Syphilis rates in Eastern Europe increased dramatically after the collapse of the Soviet Union (USSR), linked to changes in health infrastructure, sexual behavior and the emergence of the HIV/AIDS pandemic [2]. As a notifiable disease in Eastern Europe, syphilis is subject to surveillance reports, however the most recent review of the topic presented data only until 2005 [2].

Globally, the incidence of syphilis is an estimated 12 million new cases annually and WHO estimates that the majority of new syphilis cases are in Southern Asia and Sub-Saharan Africa. The effects of syphilis are far reaching, as an estimated 6.2% and 9.7% of global neo-natal deaths and stillbirths respectively are caused by untreated maternal syphilis. There are several controversies regarding syphilis globally. Such as, debate of what extent men who have sex with men (MSM) and HIV infected individuals influence infection dynamics. It was recently shown that MSM had a 140 times greater prevalence than their heterosexual counterparts in New York City and a separate study reported a 77 times greater prevalence of syphilis in those infected with HIV [3]. Furthermore, as the causative agent cannot be cultured or genetically manipulated, it is unclear what, if any, drug resistance is present globally and also how HIV co-infection affects the clinical manifestations of the disease is yet unclear [1]. Within Europe, strong surveillance data are available within the European Union (EU) community, however out with the economic area surveillance is commonly on a case notification basis that has been criticized for its accuracy [2]. As such, estimating the burden of disease regionally is more problematic.

This review is aimed at estimating a regional burden of syphilis in Eastern Europe. The data extracted from the chosen papers will be standardised to achieve a mean incidence per 100 000 people per year. These rates will then be combined to provide an estimated regional burden and a population weighted regional burden of disease based on the standardised incidences of all 7 studies. As syphilis rates have previously been markedly different between countries this review will also provide an adjusted estimate based on the geographical subdivision of Eastern Europe into 4 regions to compliment the gross and weighted estimates (**Figure 1**).

**Figure 1:**
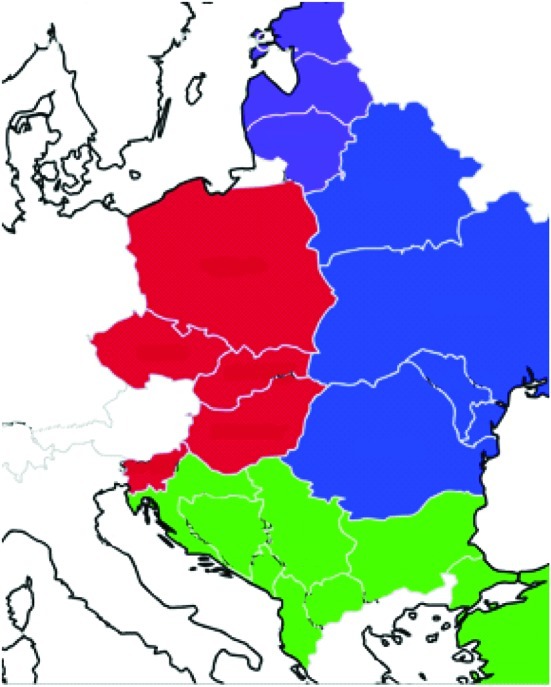
Eastern Europe as subdivided into Northern (purple), Eastern (blue), Southern (green) and Western (red) regions, for the purposes of this review.

## METHODS

### Search strategy

**Figure 2:**
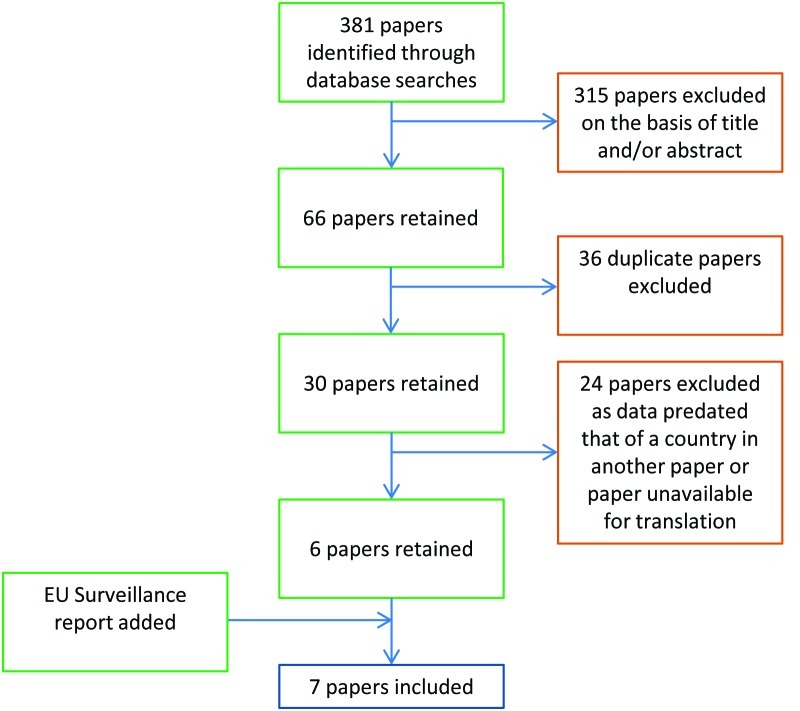
Search strategy.

The following databases were searched (**Figure 2**):

1) Web of Knowledge – 2000 onwards, using topic search terms “Syphilis and “Eastern Europe” or specific country name;

2) PubMed – 2000 onwards, using title/abstract search terms “Syphilis and “Eastern Europe” or specific country name;

3) Google Scholar Medical Database – 2000 onwards, using title search terms “Syphilis and “Eastern Europe” or specific country name.

Specific country name included all 19 countries included in study separated by Boolean operator ‘OR’. As Google Scholar has no keyword search, a title search was performed.

### Inclusion criteria

The papers resulting from our search strategy (n = 381) were first screened by title, with potentially suitable papers having their abstracts screened for any suitable data regarding syphilis (eg, new cases, incidence rates or epidemiological trends). Papers which excluded significant population demographics (eg, prison populations) or only included parts of the population (eg, blood donors, sex workers or young people) were excluded due to the inherent bias and the effect this may have on the published data were compared to the incidence for the population as a whole. Furthermore, papers only reporting epidemiological data for certain forms of the disease (eg, congenital syphilis) were also excluded as total syphilis incidence cannot be inferred from this data. Single studies or surveillance reports were treated preferentially. Reviews were analyzed and those that included appropriate data had their list of references hand-checked and, where possible, the original study was sourced for inclusion. If the original papers or reports were no longer available, or not available in English or for translation, the data presented in the review were included, as was the case for Belarus and Turkey [4,5]. Latvian, Bulgarian, Czech, Estonian, Slovak and Slovenian data were collected from the latest EU surveillance report by the latest surveillance report from the European Center for Disease Prevention and Control (ECDC) [6]. Due to the fact that the incidence of syphilis increased from 1990 but decreased markedly in most countries until a relative stagnation in 2004–2005, data from 2005 onwards were most sought after [2]. Only a single included study pre-dates this turning point, as no other single study was available for Turkey. Where multiple studies for the same country were available, the most recent data was used and the other studies were excluded. This search strategy returned 7 papers that provided data for inclusion, representing 13 countries.

### Data extraction

Data extraction from the papers differed based on the information provided. The selected papers all reported either an incidence rate per 100 000 person-years or the number of cases notified at national level across a set time period, 1–3 years for all included studies. In the event of case notifications, this data was compared with the population data available from the World Bank for the year(s) in question and this was extrapolated into an incidence rate [7]. Any study that directly reported an incidence rate had its population size analyzed using the same means and was then back transformed into a case notification rate. This allowed more accurate approximation of the sub-regional disease burden.

## RESULTS

Two of the studies included in this review were periodical surveillance reports – one at national level and the other at multinational level, providing case notifications and incidence rates for each of the countries within the EU and European Free Trade Association (EFTA) [6,8]. Two reviews were also included for analysis in this review [4,5]. The Turkish study was the only available data for the country and as such just met the inclusion criteria as the data were from 2000 [5]. The Belarusian review was focused on congenital syphilis; national data for all forms was also supplied but without an original source paper that could be analyzed independently [4]. The remaining 3 included papers were case reports at a national level [9-11], which provided extractable data from which a national incidence could be inferred (**Table 1****, ****2** and **3**).

**Table 1:** Non-standardised measures of syphilis incidence as they were reported in the studies included for analysis

Reference	Country	Disease measurement	Non-standardised value
Harxhi et al, 2010 [9]	Albania	Number of cases	37 cases
Pankratov et al. 2006 [4]	Belarus	Incidence rate	41 cases per 100 000
European Centre for Disease Control, 2011 [6]	Bulgaria	Cases and incidence rate	419 cases and 5.5 cases per 100 000
European Centre for Disease Control, 2011 [6]	Czech Republic	Cases and incidence rate	686 cases and 6.6 cases per 100 000
European Centre for Disease Control, 2011 [6]	Estonia	Cases and incidence rate	71 cases and 5.3 cases per 100 000
Ponyai et al., 2011 [10]	Hungary	Cases	2052 cases
European Centre for Disease Control, 2011 [6]	Latvia	Cases and incidence rate	223 cases and 10 cases per 100 000
European Centre for Disease Control, 2011 [6]	Lithuania	Cases and incidence rate	326 cases and 9.7 cases per 100 000
Majewski & Rudnicka, 2007 [11]	Poland	Incidence rate	2.46 cases per 100 000
Romanian Centre for prevention and control of communicable disease, 2008 [8]	Romania	Number of cases	4887 cases
European Centre for Disease Control, 2011 [6]	Slovakia	Cases and incidence rate	296 cases and 5.5 cases per 100 000
European Centre for Disease Control, 2011 [6]	Slovenia	Cases and incidence rate	47 cases and 2.3 cases per 100 000
Akturk et al., 2009 [5]	Turkey	Incidence rate	4.95 cases per 100 000

**Table 2:** Case definitions of syphilis cases from included studies

Country	Case definition
Albania [9]	Diagnosis of syphilis during time period leading to inclusion in national surveillance data.
Belarus [4]	From review paper, case definition given only as cases of syphilis per 100 000 population.
Bulgaria [6]	Diagnosis of syphilis during time period leading to inclusion in national and then EU surveillance data
Czech [6]	Diagnosis of syphilis during time period leading to inclusion in national and then EU surveillance data
Estonia [6]	Diagnosis of syphilis during time period leading to inclusion in national and then EU surveillance data.
Hungary [10]	Clinician diagnosis via direct detection of pathogens, results of serological tests and clinical picture.
Latvia [6]	Diagnosis of syphilis during time period leading to inclusion in national and then EU surveillance data
Lithuania [6]	Diagnosis of syphilis during time period leading to inclusion in national and then EU surveillance data
Poland [11]	Case reports referred to Public Health department.
Romania [8]	Cases detected from national screening procedures.
Slovakia [6]	Diagnosis of syphilis during time period leading to inclusion in national and then EU surveillance data
Slovenia [6]	Diagnosis of syphilis during time period leading to inclusion in national and then EU surveillance data
Turkey [5]	Diagnosis of syphilis during time period leading to inclusion in national surveillance data.

**Table 3:** Summary of included studies

Country	Reference	Study type	Study time period	Study location	Incidence rate (cases/100 000)
Albania	Harxhi et al. 2010 [9]	Case reports	1997-2005	Tirana, Albania	1.15
Belarus	Pankratov et al. 2006 [4]	Review	1996-2004	Belarus (National)	41
Bulgaria	European Centre for Disease Control 2011 [6]	Surveillance report	2009	Bulgaria (National)	5.5
Czech	European Centre for Disease Control 2011 [6]	Surveillance report	2009	Czech (National)	6.6
Estonia	European Centre for Disease Control 2011 [6]	Surveillance report	2009	Estonia(National)	4.2
Hungary	Ponyai et al. 2010 [10]	Case reports	2005 – 2008	Department of Dermatology, Venereology, Semmelweis University	5.1
Latvia	European Centre for Disease Control 2011 [6]	Surveillance report	2009	Latvia (National)	7.3
Lithuania	European Centre for Disease Control 2011 [6]	Surveillance report	2009	Lithuania (National)	9.7
Poland	Majewski and Rudnicka 2007 [11]	Case reports	2005-2006	Warsaw, Poland (National)	2.46
Romania	Institutul de Sanatate Publica 2008 [8]	Surveillance report	2007	Romania (National)	25.71
Slovakia	European Centre for Disease Control 2011 [6]	Surveillance report	2009	Slovakia (National)	5.5
Slovenia	European Centre for Disease Control 2011 [6]	Surveillance report	2009	Slovenia (National)	2.3
Turkey	Asturk et al. 2001 [5]	Surveillance review	1991-2000	Turkey (National)	4.95

The Eastern European region has a wide variance of syphilis incidence rates between its member countries based on the findings of this review (**Figure 2**). The maximum reported was 41/100 000, as reported in Belarus in 2004 [4], and the minimum observed incidence was 1.15 /100 000 as reported in Albania in 2005 [9]. Thus, the ratio of these two most extreme incidences was 36:1, with a range between the values of 39.85 cases/100 000. The unweighted mean for the data was an incidence of 9.34/100 000, most similar to the situation in the Lithuania in 2009 [6]. The corresponding median value was 5.5/100 000, with the 25th and 75th percentiles for the data being 4.2 and 7.3, respectively. Hence an interquartile range of 3.1 was observed. From the collective data set the estimated unweighted burden of syphilis in Eastern Europe is 9.34 new cases per 100 000 population per year (95% confidence interval (CI) 2.50–16.19), which equates to 24 073 (95% CI 6441–41710) cases in 2011, based on the 257.63 million people living in Eastern Europe at that time [7]. When the reported incidence rates were weighted against the total population at risk reported in the studies, the weighted mean for the regional burden of syphilis is 8.84 cases per 100 000 person-years (**Table 4**).

**Table 4:** Summary of results*

Statistic	Value
Mean incidence	9.3/100 000 (95% CI 2.50–16.19)
Median incidence	5.5/100 000
Maximum reported incidence	41.0/100 000
Minimum reported incidence	1.2/100 000
Max/min ratio	36:1
Range	39.9
25th percentile	4.2/100 000
75th percentile	7.3/100 000
Inter-quartile range	3.1
Weighted mean incidence	8.8/100 000
Geographically adjusted weighted mean	12.7/100 000

*Incidence is expressed in person-years, with 95% confidence (CI) interval.

For the proposed geographically subdivided estimate, the incidence rates of the Northern, Eastern, Southern and Western regions were 7.85/100 000, 30.32/100 000, 4.85/100 000 and 3.76/100 000 person-years, respectively. Using population data to extrapolate the case notifications from this incidence data, the geographically sub-divided estimate of case notifications is 32 597 cases in 2011, giving a geographically adjusted standardised rate of 12.65/100 000 person-years.

## DISCUSSION

Incidence rates have proven to be extremely heterogeneous across the 19 member states of the region, which was captured in the wide confidence interval around the unweighted mean. The geographically divided weighted mean was carried out to approach this issue. However, ideally the data for each country would be included in such an estimate, thus negating the need to estimate incidences for large countries such as the Ukraine based around the previous incidence patterns of their neighboring countries. Furthermore, the inclusion of a 95% confidence interval around the weighted mean would have been preferable.

The heterogeneity in incidence rates could also in part be due to differences in population structure. The studies only reported cases or rates for whole populations without age-group stratification. As all people are susceptible to syphilis infection the entire population are included in calculating incidence rates, it is likely that age, sex and behavior all act as confounding variables for the presented estimates [2]. Countries with an aging population or high percentage of children would likely have lower syphilis rates than a population with a greater percentage of its citizens at their sexual peak. It is well documented that birth rates have decreased in Eastern Europe since the end of the USSR and this may account for some variation in the rates between countries, but any further analysis of this issue was outside the scope of this review [2].

While EU surveillance data shows syphilis rates stabilizing post-2004, those countries outside the surveillance can be thought of as information gaps. One area of concern regarding data accuracy is Belarus. The country has not embraced a unified European outlook like many of its neighbors. Consequently, it is not included in the EU/EFTA surveillance data. The state of the health infrastructure is reflected in its reporting of the highest incidence rate. Belarusian syphilis rates reached their peak in 1996, with an incidence rate of 209.7 cases per 100 000 person-years, compared to between 2 and 6 cases per 100 000 in Poland, Hungary and the Czech Republic [2]. As the data included in this study is from 2004, this rate may not be representative of the true incidence rate anymore. Similar assumptions could be assigned to the estimated incidence in the Ukraine.

A suggestion for future research would be to conduct epidemiological surveillance into the incidence of syphilis in the countries of the former Soviet Union. The most recent review of syphilis in Eastern Europe stated that the incidence rate of syphilis within the Russian Federation was >50 cases per 100 000 person-years in 2005 [2]. Another review into the incidence of HIV and syphilis in Central Asian countries was carried out in 2003, returning incidence rates for 2002 of 122, 55, 12.7 and 25.8 per 100 000 person-years in Kazakhstan, Kyrgyzstan, Tajikistan and Uzbekistan, respectively [12]. Such high incidence rates are indicative that these countries experienced the same epidemics of sexually transmitted infections (STI) which followed the collapse of the Soviet Union in other countries. From this data it can be seen that the incidence rates within the former Soviet Union are generally higher than for non-Soviet Eastern European countries, although the North-Eastern European region returned incidences closer to their EU neighbors. Reasons for this may include changing demography and migration patterns within the region, eg, more young people migrating to cities to search for employment. It should be noted that the Central Asian data are now 10 years old and as syphilis rates in Eastern Europe have decreased substantially during this period, and so may have the Central Asian rates. However, this is still an avenue open to further research, as comprehensive data on the topic is sparse and analysis of trends may aid control and prevention efforts.

Of the papers retrieved from our literature search, several were regional estimates of syphilis incidence at a city or provincial level. While this review only included national estimates, it is important to note that regional studies are not always reflective of incidence rates at national level. Other studies have shown previously that syphilis rates can vary greatly within the provinces of a country [13] and the findings of this review concur with this assessment. An example of this is Czech Republic in 2009. Our literature search returned a paper that provided case notification data from a regional surveillance conducted in the Prague metropolitan area [14]. Population data for this region was sourced from the Czech Department of Statistics and the case notifications were transformed into an incidence rate for the region using this data [15]. The EU national surveillance rate and the regional rate were 6.6 and 10.8 cases per 100 000 person-years, respectively. As such, the regional estimate returned an incidence rate was 1.6 times that of the national estimate. Thus, future reviews into the burden of syphilis should be wary of including regional data as regional estimates may confound their results. A possible explanation for such a difference between the estimates is the demography of region. Prague, being the country’s capital, hub of tourism and economic center may vary significantly from other regions in the density of subpopulations including men who have sex with men, sex workers and people in their sexual peak.

A comparison of the 2009 ECDC surveillance report data for the 5 included countries with that for 2006 shows diverging patterns in syphilis trends. It is noted by the ECDC itself that there is likely to be significant under reporting of cases and there is varying quality in the surveillance networks in each country. In Slovenia, for example, reporting is carried out by physicians with no laboratory or hospital reporting [6]. The 3 countries for which there was a large percentage increase have typically exhibited year on year increase since 2006, which cannot be attributed to either increase in syphilis incidence, or increased surveillance capability, without more in depth understanding of the development of syphilis reporting system in each country. Furthermore, there is no way to determine how accurate the reporting is for private health services. Ultimately, surveillance accuracy will be dependent on the countries health care infrastructure and as such surveillance data from the less affluent countries should be viewed with caution. When assessing the included literature as a whole, the case definitions for syphilis were not explicit for many studies. This introduces the probability of diagnostic misclassification bias, especially when considered alongside the poor access to diagnostic technology in several countries. It can be assumed that not all cases were subjected to *Treponema* testing, serology or dark field microscopy to identify the bacteria, thus diagnoses may have been made purely on clinical presentation or non-treponemal tests, many of which have well documented specificity issues [16-19]. Random misdiagnoses, while problematic, would not be as damaging to the data’s validity as systematic misdiagnosis based on altered clinical presentation due to co-infection with HIV for example, which has been the subject of several other studies [16-17].

### Conclusions

The incidence of syphilis in the EU 15 countries was 2.78/100 000 in 2007 [6]. When compared to our geographically subdivided estimate of 12.51/100 000 for the Eastern Europe, this highlights the great disparity in the control of sexually transmitted infections between the regions. Healthcare planning must strengthen treatment options for HIV and syphilis, as both diseases act as risk factors for each other. Reductions in HIV prevalence are likely to have a beneficial effect on syphilis prevalence and outcomes. However, syphilis infection increases chances of HIV transmission by 7 times and thus treatment can be seen as a way to reduce HIV prevalence in high risk groups [17]. Possible incentives for progress in this area are commitments to the UN Millennium Development Goals, of which HIV reduction and reducing child mortality, in this case due to congenital syphilis, are both included [17].

Investing in health infrastructure and surveillance capability would strengthen the control of syphilis. Increased knowledge of transmission dynamics would allow targeting of specific regions or subpopulations where the burden of disease is higher. One area that health policy research in Eastern Europe should address is transparency in the state of health provision and the percentage of health care provided by private services. Previous reviews have highlighted the impact of these services on STI control, but have been unable to quantify their effect [2]. Thus, this can be considered a confounder to available disease estimates including the ECDCs surveillance [6].

Current opinion suggests funding should be focused on extending the availability and efficacy of the rapid *Treponema* tests [18,19] and the extension of education programs [20]. Enhancement of the availability of rapid testing would reduce the reliance on more traditional serological and microscopy methods. Those traditional methods require transport of blood to centralised facilities and the maintenance of a “cold chain” to ensure validity of the test [19]. Furthermore, vaccine development from its current stage of testing in rabbit models should receive support, because mass vaccination campaigns have the potential to significantly to reduce the transmission potential of syphilis [19].
